# Supplementary material for: Sustained-input switches for transcription factors and microRNAs are central building blocks of eukaryotic gene circuits
Source: Genome Biol. 2013 Aug 23;14(8):R85. doi: 10.1186/gb-2013-14-8-r85 (PMC4054853; doi:10.1186/gb-2013-14-8-r85)
Supplement: Additional file 5 — HTML Browsable Motif Output. Zipped folder containing all WaRSwap and FANMOD motif output, viewable in a web browser. [file gb-2013-14-8-r85-S5.ZIP › HTML_browsable_motif_output/FANMOD_ath_tair9/sigs_fanmodm-2000.pvals.heatmaps.html/motif_id_46_011101002_tftype_ath_upstream_-2000_0.html]

```
BG_MODEL = FANMOD
MOTIF_ID = 46_011101002
TF_TYPE = ath
UPSTREAM = -2000_0


PVals
FN_0.2	FN_0.4	FN_0.6	FN_0.8
dg_60.genes	0.135	0.001	0.048	0.585
dg_70.genes	0.136	0.003	0.065	0.558
dg_80.genes	0.126	0.001	0.058	0.573

ZScores
FN_0.2	FN_0.4	FN_0.6	FN_0.8
dg_60.genes	1.11	2.96	1.598	-0.891
dg_70.genes	1.094	2.893	1.51	-0.862
dg_80.genes	1.127	3.084	1.584	-0.9

StDevs
FN_0.2	FN_0.4	FN_0.6	FN_0.8
dg_60.genes	23381.601	23755.306	5771.006	659.722
dg_70.genes	23188.502	24714.867	6029.064	652.299
dg_80.genes	23095.228	23153.625	5781.324	644.082
```
